# In Vitro and In Vivo Antioxidant Activity of the New Magnetic-Cerium Oxide Nanoconjugates

**DOI:** 10.3390/nano9111565

**Published:** 2019-11-04

**Authors:** Ioana-Andreea Turin-Moleavin, Adrian Fifere, Ana-Lacramioara Lungoci, Irina Rosca, Adina Coroaba, Dragos Peptanariu, Valentin Nastasa, Sorin-Aurelian Pasca, Andra-Cristina Bostanaru, Mihai Mares, Mariana Pinteala

**Affiliations:** 1 Centre of Advanced Research in Bionanoconjugates and Biopolymers Department, “Petru Poni” Institute of Macromolecular Chemistry, 41A Grigore Ghica-Voda Alley, 700487 Iasi, Romania; moleavin.ioana@icmpp.ro (I.-A.T.-M.); lungoci.lacramioara@icmpp.ro (A.-L.L.); rosca.irina@icmpp.ro (I.R.); adina.coroaba@icmpp.ro (A.C.); peptanariu.dragos@icmpp.ro (D.P.); 2 Laboratory of Antimicrobial Chemotherapy, “Ion Ionescu de la Brad” University of Agricultural Sciences and Veterinary Medicine, 8 Sadoveanu Alley, 700489 Iasi, Romania; vnastasa67@gmail.com (V.N.); spasca@uaiasi.ro (S.-A.P.); acbostanaru@gmail.com (A.-C.B.); mihaimares@fungi.ro (M.M.)

**Keywords:** core-shell magnetic nanoparticles, antioxidant activity, free radical scavengers, cerium oxide nanoparticles

## Abstract

Background. Cerium oxide nanoparticles present the mimetic activity of superoxide dismutase, being able to inactivate the excess of reactive oxygen species (ROS) correlated with a large number of pathologies, such as stents restenosis and the occurrence of genetic mutations that can cause cancer. This study presents the synthesis and biological characterisation of nanoconjugates based on nanoparticles of iron oxide interconnected with cerium oxide conjugates. Methods. The synthesis of magnetite-nanoceria nanoconjugates has been done in several stages, where the key to the process is the coating of nanoparticles with polyethyleneimine and its chemical activation-reticulation with glutaraldehyde. The nanoconjugates are characterised by several techniques, and the antioxidant activity was evaluated in vitro and in vivo. Results. Iron oxide nanoparticles interconnected with cerium oxide nanoparticles were obtained, having an average diameter of 8 nm. Nanoconjugates prove to possess superparamagnetic properties and the saturation magnetisation varies with the addition of diamagnetic components in the system, remaining within the limits of biomedical applications. In vitro free-radical scavenging properties of nanoceria are improved after the coating of nanoparticles with polyethylenimine and conjugation with magnetite nanoparticles. In vivo studies reveal increased antioxidant activity in all organs and fluids collected from mice, which demonstrates the ability of the nanoconjugates to reduce oxidative stress. Conclusion. Nanoconjugates possess magnetic properties, being able to scavenge free radicals, reducing the oxidative stress. The combination of the two properties mentioned above makes them excellent candidates for theranostic applications.

## 1. Introduction

In recent decades, nanotechnological applications open the way to the concept of nanomedicine. Also, with the evolution of experimental nanomedicine, the theranostic nanomedicine term was developed using an integrated therapeutic system capable of functioning in a wide range of applications that allow group diagnosis and early stage disease treatment [1,2]. 

With this idea, nanoparticles possessing different properties are beginning to be combined into new materials by their conjugation to obtain products that are suitable for complex applications in theranostic nanomedicine. In particular, core-shell magnetic nanoparticles (MNPs) are combined with gold (Au) [3], silver (Ag) [4] and titanium oxide (TiO_2_) [5] nanoparticles in order to obtain nanoconjugates with specific properties of the two types of nanoparticles. In this respect, core-shell magnetic nanoparticles (MNPs) can be successfully used as agents for transport and delivery (at the desired site) of an antioxidant drug capable of inactivating reactive oxygen species (ROS) and/or reactive nitrogen species (RNS), hindering the side effects that may occur, such as DNA mutations and lipid peroxidation [6,7,8,9]. Over-production of reactive species limits the antioxidant defences of the living body that causes the occurrence of numerous disorders, such as cancer, as well as cardiovascular, inflammatory and neurodegenerative diseases [8].

MNPs have drawn attention, due to their particular specific properties (stability, possibility of functionalisation, superparamagnetic properties and the fact that they can be guided and accumulated in tissues of choice), offering the opportunity to be used in biology [10,11,12]. Such complex entities are already applied as heat-inducing vectors for the treatment of solid cancerous tumours by intracellular hyperthermia, or as drug delivery carriers after their loading with different bioactive agents [13,14,15,16,17]. In addition to numerous therapeutic applications, due to their magnetic properties, magnetite nanoparticles are used as magnetic resonance imaging agents [15], playing an important role in cardiovascular applications [18], therefore fulfilling an important role in medical diagnosis. Consequently, multifunctional magnetic nanoparticles can provide new insights into the development of theranostic medicine [15,19].

There is a wide variety of antioxidants with biomedical applications, able to inactivate free radicals, including a large number of classes of natural compounds, as well as organic and inorganic synthesis compounds [20,21,22]. Among inorganic antioxidants, nowadays, cerium oxide nanoparticles (CeNP) are starting to exert a high interest due to their free radical scavenging properties, playing the role of radical scavenger with neuroprotective, radioprotective and anti-inflammatory properties [23]. Cerium oxide nanoparticles of 2–6 nm have proven to demonstrate superoxide dismutase (SOD) mimetic activity, emphasised by ferricytochrome C assay [24]. Their antioxidant properties make them highly effective as scavengers for the superoxide anions or reactive nitrogen species (RNS) present in biologic milieus [25,26]. The surface oxidation state of cerium oxide nanoparticles plays an important role in their redox properties, those that are directly related to the Ce^3+^/Ce^4+^ ratio and the efficiency of the interchanging capacity between Ce^3+^ and Ce^4+^ on their surfaces, which are reflected in the nanoparticles’ sizes as well [25,26].

Under these circumstances, our aim was to engineer a complex structured nanoentity able to deliver antioxidant chemical species in a pharmacologically-controlled and spatially-guided manner. More specifically, the purpose of this study is to prepare and to characterise, for the first time, a nanosize cargo-complex, having a magnetic core and a polyethyleneimine (PEI)-based shell, able to load and to deliver precise amounts of the radical scavengers of cerium oxide nanoparticulate type, being spatially guided in the human body (by means of a magnetic field) and supplying species capable of the annihilation of active radical species. Hyperbranched polyethylenimine (PEI) is a well-known and intensively studied biocompatible polymer, and it is tested and used in bio-applications, such as being a transfection reagent for drug delivery systems or tissue culture [27,28,29]. As recently demonstrated by our group, 1.8 kDa-branched PEI is able to form a shell around the magnetite nanoparticle, through electrostatic interactions, and quickly facilitating the loading of the protocatechuic acid, even of its inclusion complex with sulfobutylether-β-cyclodextrin, to deliver the natural antioxidant able to scavenge free radicals [6].

The physical-chemical characterisation of the obtained nanoparticles was done by Fourier-transform infrared spectroscopy (FT-IR), Raman, energy-dispersive X-ray spectroscopy (EDX), X-ray photoelectron spectroscopy (XPS), transmission electron microscopy (TEM), dynamic light scattering (DLS) and magnetisation measurements. 

The antioxidant and radical scavenging properties were investigated in vitro using the popular method based upon the use of stable free radical 1,1-diphenyl-2-picryhydrazyl (DPPH), and in vivo by measuring the total antioxidant capacity after administration in mice.

## 2. Materials and Methods

### 2.1. Materials

Chemical reagents were of analytical grade and used as received without further purification. Cerium(III) nitrate hexahydrate (Ce(NO_3_)_3_∙6H_2_O), ferric chloride (FeCl_3_∙6H_2_O), ferrous chloride (FeCl_2_∙4H_2_O), branched polyethylenimine of 1.8 kDa, 25% glutaraldehyde aqueous solution (GA), 25% ammonium solution and 2,2-diphenyl-1-picrylhydrazyl (DPPH) were purchased from Sigma-Aldrich; normal human dermal fibroblasts (NHDF) from PromoCell; CellTiter 96^®^ AQueous One Solution Reagent, containing tetrazolium compound [3-(4,5-dimethyl-2-yl)-5-(3-carboxymethoxyphenyl)-2-(4-sulfophenyl)-2H-tetrazolium, inner salt (MTS)] and electron coupling reagent (phenazine ethosulfate; PES) from Promega; alpha-MEM medium and 1% Penicillin-Streptomycin-Amphotericin B mixture from Lonza; 10% fetal bovine serum (FBS) from Gibco; T75 cell culture flasks from Corning; Antioxidant Assay Kit CS0790 from Sigma-Aldrich (St. Louis, MO, USA). 

### 2.2. Characterisation Techniques

The Fourier transformed infrared (FTIR) spectra (400–4000 cm^−1^) from KBr pellet were recorded using a Bruker Vertex 70 FTIR instrument (Billerica, MA, USA), in transmission mode, at room temperature.

The Raman spectra were registered using a Renishaw inVia confocal microscope equipped with a He–Ne laser at 632.8 nm (17 mW) and a CCD detector coupled to a Leica DM 2500 M microscope. The range of vibrational frequencies is from 100 to 3200 cm^−1^. All measurements were performed in backscattering geometry using a 50× objective with a numerical aperture of 0.75. The Raman measurements were performed at room temperature and atmospheric pressure. Spectral manipulations were performed with the WiRE 3.2 software (Renishaw, Kingswood, UK).

The composition analysis was studied with the Quanta 200 microscope (FEI Company, Brno, Czech Republic) equipped with an Energy Dispersive X-Ray system (EDX) for qualitative and quantitative analysis and elemental mapping. This technique of elemental analysis is based on the generation of characteristic X-rays that reveals the presence of chemical elements in the samples. The EDS X-Ray detector measures the relative abundance of emitted X-rays versus their energy.

X-ray photoelectron spectroscopy (XPS) was performed on a KRATOS Axis Nova (Kratos Analytical, Manchester, United Kingdom), using AlKα radiation, with 5 mA current and 15 kV voltage (75W), and a base pressure of 10^−8^ to 10^−9^ Torr in the sample chamber. The XPS survey spectra for the samples was collected in the range of −10 ÷ 1200 eV with a resolution of 1 eV and a pass energy of 160 eV. The high resolution spectra for all the elements identified from the survey spectra were collected using a pass energy of 20 eV and a step size of 0.1 eV. Data were analysed using the Vision Processing software (Vision2 software, version 2.2.10, Kratos Analytical, Manchester, UK). The binding energy of the C1s peak was normalised to 284.6 eV.

The particle size distribution and zeta potential of the prepared nanoparticles were determined by dynamic light scattering (DLS) using a Delsa Nano C Submicron Particle Size Analyser (Beckman Coulter, Inc., Fullerton, CA, USA). The samples were dispersed before measurements by ultrasonication, and the measurements were recorded at room temperature in flow cell for both size and zeta potential measurements.

Transmission electron microscopy (TEM) studies were carried out with a Hitachi High-Tech HT7700 Transmission Electron Microscope (Tokyo, Japan) that operated at a 100 kV accelerating voltage in High-Contrast Mode. The samples were prepared by placing a droplet of aqueous suspension of nanoparticles on carbon-coated copper grids with 300-mesh sizes, then allowing the solvent to evaporate at room temperature. Statistical data of the size distributions were obtained using the Image J software by measuring 100 nanoparticles.

UV-Vis measurements were performed on a Lambda 35 apparatus (Perkin Elmer Inc., Waltham, MA, USA).

Measurements of magnetisation were carried out with a Vibrating Sample Magnetometer, from MicroMag (Princeton Measurements Corporation, Princeton, NJ, USA), at room temperature, in solid state.

### 2.3. Synthesis of Nanoparticles

#### 2.3.1. Synthesis of Core-Shell Cerium Oxide Nanoparticles (CeNP) Covered with PEI Crosslinked (CePEI-GA)

The synthesis was performed in two stages.

(a) Synthesis of CeNP coated with PEI (CePEI) was performed following a previously reported method with some modifications [23]. Briefly, 10 mL (1M) solution of polyethylenimine (PEI) was added under magnetic stirring to a 30 mL (25%) solution of NH4OH. Then, 5 mL (1M) solution of Ce(NO_3_)_3_∙6H_2_O was added drop wise, under magnetic stirring, to the previous solution. The reaction took place for 24 h at room temperature. The resulting yellow precipitate was separated by centrifugation and washed several times with water in order to remove the NH_4_OH and unbounded PEI chains. These CeNPs covered with PEI are stabilised by physical forces.
(b) Synthesis of core-shell CeNP coated with PEI crosslinked by GA (CePEI-GA). In order to stabilise the PEI shell onto the CePEI, PEI was crosslinked with GA. Briefly, 24 mL (24%) GA solution was added drop wise, under magnetic stirring, to 36 mL (10 mg/mL) of an aqueous solution of previously synthetised CePEI nanoparticles. Reaction took place in 3 h, at room temperature, and the excess of GA was removed by washing the product several times with water. To insure that GA is totally reacted and all of the aldehyde groups are inactivated, 20 mL solution of PEI (10% in water) was added and reacted overnight. The final product was washed with water several times in order to remove the unreacted PEI, and then it was stored as a solution at 4 °C.

#### 2.3.2. Synthesis of MNP Covered with PEI (MPEI)

The synthesis was performed in two steps.

(a) Synthesis of bare magnetic nanoparticles (MNPs) was performed following the classical method of co-precipitation without modifications [30]. Briefly, 20 mL (25%) ammonium solution was added to a mixture of ferric and ferrous salts aqueous solutions, under nitrogen flow and mechanical stirring, having the Fe^2+^/Fe^3+^ = 1/2 molar ratio. The mixture became black immediately, and was stirred for another 30 min at 70 °C under nitrogen flow. The obtained MNPs were washed several times with water and ethanol. The bare MNPs were stored in ethanol at 5 °C in the refrigerator prior to use.
(b) Preparation of core-shell MNP coated with PEI (MPEI) was done by a previously reported method, without any modification [6]. In brief, 10 mL of PEI (10%, 1.8 kDa) solution was added to 8 mL suspension of fresh MNP (50 mg/mL) in deionised water, sonicated for 5 min and stirred for 24 h. Excess of PEI was removed by washing the product five times with water using magnetic decantation. The MPEI conjugates can be stored for a long period of time in deionised water before use in subsequent reaction steps.

#### 2.3.3. Synthesis of Hybrid Nanoparticles (MCePEI-GA)

(a) Crosslinking of the PEI layer of MPEI nanoparticles and activation with aldehyde–aldehyde groups. PEI shell of MPEI was reticulated with GA by the same protocol presented above for CePEI-GA, except that, after the GA elimination from the supernatant, aldehyde–aldehyde groups from the PEI shell of MPEI reacted with GA were not deactivated by a subsequent reaction with PEI. At this stage, MNP remains covered with PEI crosslinked with GA and activated by inherently remaining free aldehyde groups. This product cannot be stored for a long time, and should be used quickly in subsequent reactions.
(b) Synthesis of magnetite-nanoceria nanoparticles. 10 mL liquid dispersion of freshly activated MPEI with GA (100 mg) was added to 10 mL CePEI-GA of 100 mg, prior sonicated (1 min) and allowed to react under stirring at room temperature for 24 h. Afterwards, in order to deactivate the remaining aldehyde groups, the reaction was stopped by adding 20 mL of 10% PEI (1.8 kDa), and allowed to react for another 24 h in the same conditions. After these procedures, only the fraction that separated magnetically was collected. NPs which resulted from magnetic separation were washed with water five times in order to remove the unreacted PEI and were then stored as dispersions in water at 4 °C.

### 2.4. In Vitro Studies

#### 2.4.1. Ex-Vivo Radical Scavenging Activity

In order to evaluate the antioxidant activity of the nanoparticles, the bleaching of the 1,1-diphenyl-2-picryl hydrazyl (DPPH) radical was used. Briefly, a stock solution of 2 mg/mL of the nanoparticles in methanol was prepared and then diluted to obtain different concentrations (7.81 ÷ 1000 µg/mL). Proper volumes of the diluted solutions were mixed with an equal volume of methanolic DPPH solution (0.05 mg/mL); the resulted solutions were incubated in the dark at room temperature (25 °C) for 2 h. After incubation, the absorbance was read at 517 nm. Inhibition percentages were calculated using Equation (1):

(1) $$Inh\%=\;\frac{A_{C}-A_{S}}{A_{C}}100$$

where, *A_c_* is the absorbance at 517 nm of control sample (DPPH), and *A_S_* the absorbance at 517 nm for the sample with antioxidants containing nanoceria and DPPH. Plotting Inh% versus DPPH concentration, the IC_50_ value can be determined, designating the concentration of the sample required to scavenge 50% of the DPPH free radicals [31]. All measurements were made in triplicate, with average (mean) values being presented in the article.

#### 2.4.2. Cytotoxicity Assay

To assess the cytotoxic effects of the nanoparticles, mitochondrial activity was investigated by MTS assay performed on NHDF cells. Fibroblasts were cultivated and expanded in alpha-MEM medium supplemented with 10% foetal bovine serum and 1% Penicillin-Streptomycin-Amphotericin B (10K/10K/25 µg in 100 mL) in T75 cell culture flasks until a sufficient number of cells for experiment was obtained.

For this assay cells were seeded at the concentration of 5 × 10^3^ cells/well/100 µl in a 96-well plate and incubated at 37 °C, 5% CO_2_ under a humidified atmosphere. After 24 h, the medium was removed and a range of nanoparticle concentrations in the cell culture medium (from 0.78 to 100 µg/mL) was poured over the cells, and the plates were returned to the incubator. Cells receiving only cell culture medium were used as negative control.

After 44 h of incubation, without removing the nanoparticle solutions, 20 µl/well of CellTiter 96^®^AQueousOne Solution reagent (MTS) was added to the plates which were incubated for another 4 h at 37 °C. During this time, mitochondrial dehydrogenase converts MTS tetrazolium to a soluble formazan which has a specific absorbance at 490 nm. The absorbance of samples at 490 nm was read and recorded with a plate reader (iMark™ Microplate Absorbance Reader, Bio-Rad, Watford, UK) and absorbance values were converted to relative cell viability (%) using the formula [(A_t_ − A_b_)/(A_c_ − A_b_)] × 100 where A_t_ = absorbance value of the sample, A_c_ = absorbance value of the control and A_b_ = absorbance value of the blank. The results presented as mean ± standard deviation. The experiment was repeated three times and a minimum six replicates were used for analysis.

### 2.5. In Vivo Studies

#### 2.5.1. Animals

In order to evaluate the possible biological effects of the synthetised nanoparticles (i.e., toxic and antioxidant), in vivo tests on mice were performed. A total number of 15 adult female outbred BALB/c mice (Cantacuzino Institute, Bucharest, Romania) with a mean weight of 21 ± 0.19 g were used. The animals were divided in three groups of five mice each (groups A to C), hosted in 1500 cm^2^ polycarbonate cages, and let to acclimatise for 72 h until the day of experiment. During this period, all mice had unrestricted access to standard feed and tap water. The following microclimate conditions were assured: Temperature 22 ± 0.7 °C, relative humidity 60 ± 10%, and a cycle of 12-h light/12-h dark.

#### 2.5.2. Experimental Design

The nanoparticles (CePEI-GA and MCePEI-GA, respectively) were dispersed in saline solution, assuring equivalent concentrations of the active compound (Ce). The concentrations of the injected solutions were calculated so that the amounts of free CePEI-GA and CePEI-GA in the MCePEI-GA matrices were the same (calculated from EDX data). Thus, a final concentration of 4 μg/mL was used for CePEI-GA, while for MCePEI-GA the final concentration was 100 μg/mL.

Animals from group A received saline solution, animals from group B received CePEI-GA solution, and those from group C received MCePEI-GA solution. All solutions were injected intraperitoneally using a standard volume of 1 mL.

After 24 h, all animals were anaesthetised with isoflurane and blood samples were collected by cardiac puncture in order to obtain plasma. The blood was mixed with potassium ethylenediaminetetraacetic acid (EDTA) solution to prevent the coagulation and further processed by centrifugation at 3000 g in the first 30 min after the puncture in order to obtain plasma. This procedure was followed by euthanasia performed with injectable sodium pentobarbital and gross necropsy. Samples from the brain, spleen, and liver were collected from each animal for the antioxidant activity assay.

The study fully complies with the recommendations of Directive 2010/63/EU, and was approved by the Ethics Committee of the institution.

#### 2.5.3. In Vivo Antioxidant Tests

Total antioxidant capacity in liver, spleen, brain homogenates and blood plasma samples from untreated and treated mice was evaluated using the Trolox equivalent antioxidant capacity assay with the help of Sigma-Aldrich Antioxidant Assay Kit, catalog number CS0790 (St. Louis, MO, USA). All the measurements were made following the procedures for preparing solutions and samples described in the technical bulletin of the above-mentioned kit.

In this assay, myoglobin in its reduced state, named metmyoglobin, is oxidised by hydrogen peroxide, forming the ferryl myoglobin radical, which oxidises the 2,2′-azino-bis(3-ethyl-benzthiazoline-6-sulfonic acid (ABTS) to produce the ABTS^+^• cation radical. This radical can be measured quantitatively by spectrophotometry at a wavelength of 405 nm. The antioxidant inactivates the ABTS^+^• free radical and the absorbance at this given wavelength decreases proportionally with their concentration.

The antioxidant effect is evaluated by measuring the amount of antioxidant using Trolox as the standard. In the standard curve, Trolox was used as an antioxidant control, plotting the absorbance decay of the ABTS^+^• versus Trolox concentration. In our experiment, plasma, spleen, liver and brain homogenates samples from untreated and treated mice with MCePEI-GA or CePEI-GA play the antioxidant role due to their intrinsic antioxidant activity and the administration of cerium oxide conjugates, respectively. Therefore, the amount of antioxidants in the biological samples was expressed as a Trolox concentration (mM) obtained from the linear regression of the standard curve.

#### 2.5.4. Data Analysis

For data analysis GraphPad Prism version 7 (GraphPad Software, La Jolla, CA, USA) was used. Results are expressed as the mean value ± standard deviation obtained from three measurements.

## 3. Results and Discussion

### 3.1. Synthesis of Nanoconjugates with Antioxidant Properties

**Figure 1:**
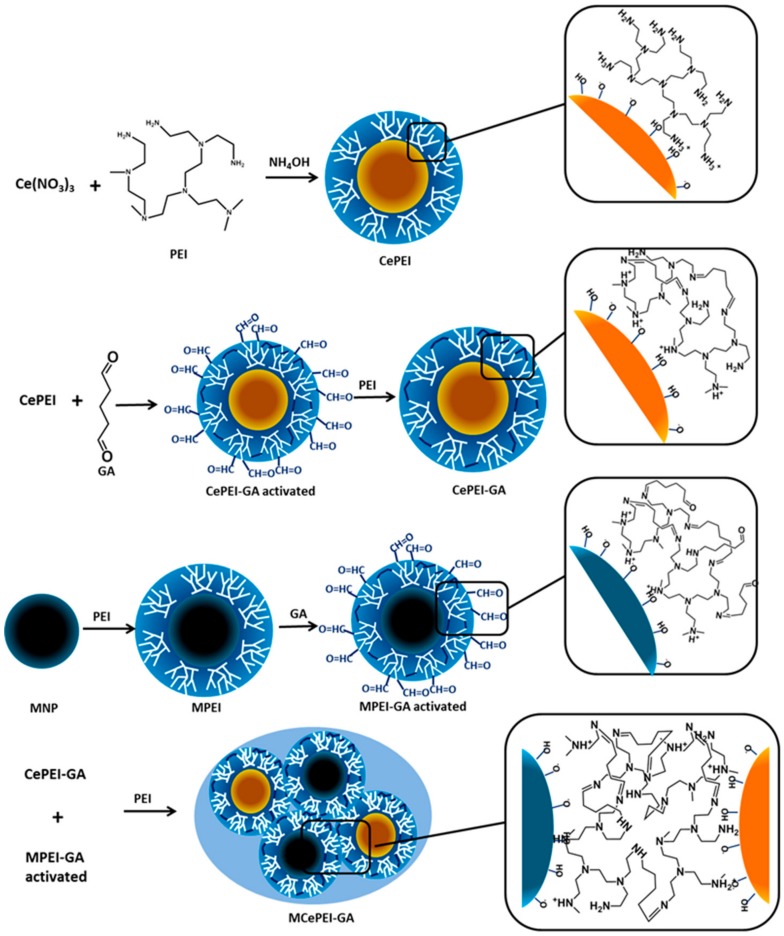
Schematic representation of nanoparticles synthesis.

The proposed design for MCePEI-GA, as a new nanoentity, was achieved starting with the synthesis of the CePEI-GA conjugate by mixing, for 24 h, the solutions of PEI at high pH and of Ce^3+^ salt, followed by the cross-linking of the PEI shell with GA in order to stabilise the CePEI nanoparticles (Figure 1). It should be mentioned that in CePEI-GA conjugates, all free aldehyde groups are annihilated by adding additional PEI. Magnetite nanoparticles (MNP) were synthetised following the classical method of co-precipitation, from a mixture of ferric and ferrous salts at high pH [11,32]. Further, based upon the previous experience [6], the fresh magnetite nanoparticles were coated with PEI by a simple mixing of the MNP aqueous dispersion with the PEI solution. At this stage the MPEI nanoparticles are stabilised by physical interactions, such as electrostatic interactions, given by the PEI polycation and MNPs with negative zeta-potential, becoming stable in all physicochemical measurements [6].

Finally, MCePEI-GA nanoconjugates were synthesised in two steps, using previously synthesised CePEI-GA and MPEI nanoparticles. In the first stage, PEI from MPEI was cross-linked with GA in a manner similar with that described in the CePEI-GA synthesis. In this case, however, after the cross-linking with GA, the PEI shell remains activated by the reactive aldehyde–aldehyde groups of GA, being ready to react with extra amine groups from the shell of CePEI-GA nanoparticles in the subsequent synthesis of MCePEI-GA hybrid nanoparticles (Figure 1). It should be noted that the cross-linking of PEI from MPEI shells was made by dispersing MPEI nanoparticles in a concentrated solution of GA, leading to the idea that the number of available amine groups from the MPEI shell is reduced enough, being consumed by GA, and as a consequence a drastically reduced tendency of agglomeration can be observed. Therefore, the reaction between the aldehyde groups grafted onto activated MPEI and the PEI from the CePEI-GA is highly favoured (Figure 1).

### 3.2. FT-IR Study of MPEI, CePEI-GA and MCePEI-GA Nanoparticles

**Figure 2:**
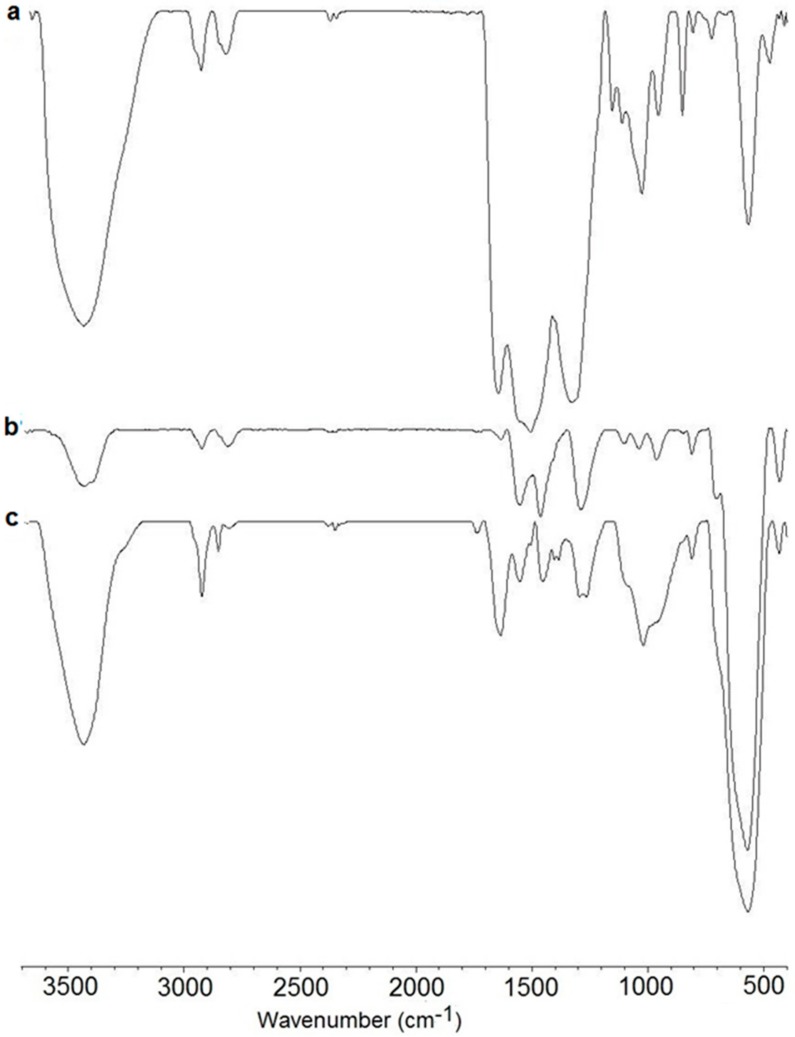
Fourier transformed infrared (FTIR) spectra of (CeNP) covered with PEI crosslinked (CePEI-GA) (**a**), MNP Covered with polyethylenimine (PEI) (MPEI) (**b**) and MCePEI-GA (**c**).

To confirm the structure of the MCePEI-GA, the FT-IR spectrum is recorded in comparison with those of the MPEI and CePEI-GA conjugates (Figure 2).

The formation of CePEI-GA nanoparticles (Figure 2a) is proven by the presence of a Ce–O stretching vibration at 565 cm^−1^ [33], while the characteristic peaks of PEI are expressed in the absorbtion bands at 3433 cm^−1^ (characteristic for –N–H stretching, superposing with –HO vibration of water from moisture), 2819–2925 cm^−1^ (–C–H stretching), 1506 cm^−1^ (–N–H bending) and 1350–1000 cm^−1^ (–C–N stretching) [34]. Moreover, the spectrum displays a distinct peak at 1645 cm^−1^, which is assigned to the stretching band of –C = N, the imine bond formation after the reaction between GA’s aldehyde groups and the amine groups of PEI [34]. The FT-IR spectrum of MPEI nanoparticles (Figure 2b) shows characteristic peaks at 570 cm^−1^, assign to Fe–O vibrations bands [6], and those more intense from PEI at 1550 cm^−1^ (–N–H bending) and 1463 cm^−1^ (–C–H bending) [6].

Taking all of these into consideration, it can be observed that the FT-IR spectrum of MCePEI-GA nanoparticles (Figure 2c) displays all the characteristic peaks of both CePEI-GA and MPEI nanoparticles, moreover being much more orderly, with two exceptions when the absorption band at 1635 cm^−1^, attributed to the –C = N bonds, is shifted to the right, as compared to those of CePEI-GA (Figure 2a,b) and the band of 565 cm^−1^ is more intense, due to its width, overlapping the characteristic bands for the Ce–O and Fe–O bonds. Finally, the sum of the signals in the MCePEI-GA spectrum confirms the formation of the hybrid system made up from the cerium oxide and iron oxide nanoparticles, joined by PEI cross-linked with GA.

### 3.3. Raman Study of MCePEI-GA

**Figure 3:**
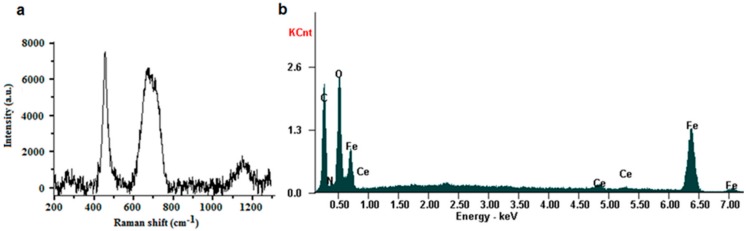
Raman spectra (**a**) and energy-dispersive X-ray spectroscopy (EDX) analysis (**b**) for the MCePEI-GA sample.

Data from Raman spectroscopy of MCePEI-GA conjugates (Figure 3a) reveals that the two types of nanoparticles are conjugated, maintaining their structural integrity and hence their physico-chemical properties [35]; these statements are supported by the presence of two strong intense bands at 460 cm^−1^ attributed to CeNP, and 670 cm^−1^ attributed to the presence of MNP nanoparticles [33].

### 3.4. EDX Characterisation

Energy-dispersive X-ray spectroscopy (EDX) was used to identify the elemental composition of the conjugate nanoparticles. In the EDX spectrum of MCePEI-GA, the signals corresponding to the iron binding energy, along with those of the cerium, can be observed (Figure 3b). Moreover, the simultaneous presence of Fe, Ce, C, O and N atoms in the spectrum confirm the existence of the characteristic elements from the MCePEI-GA conjugate.

### 3.5. XPS Measurements

**Figure 4:**
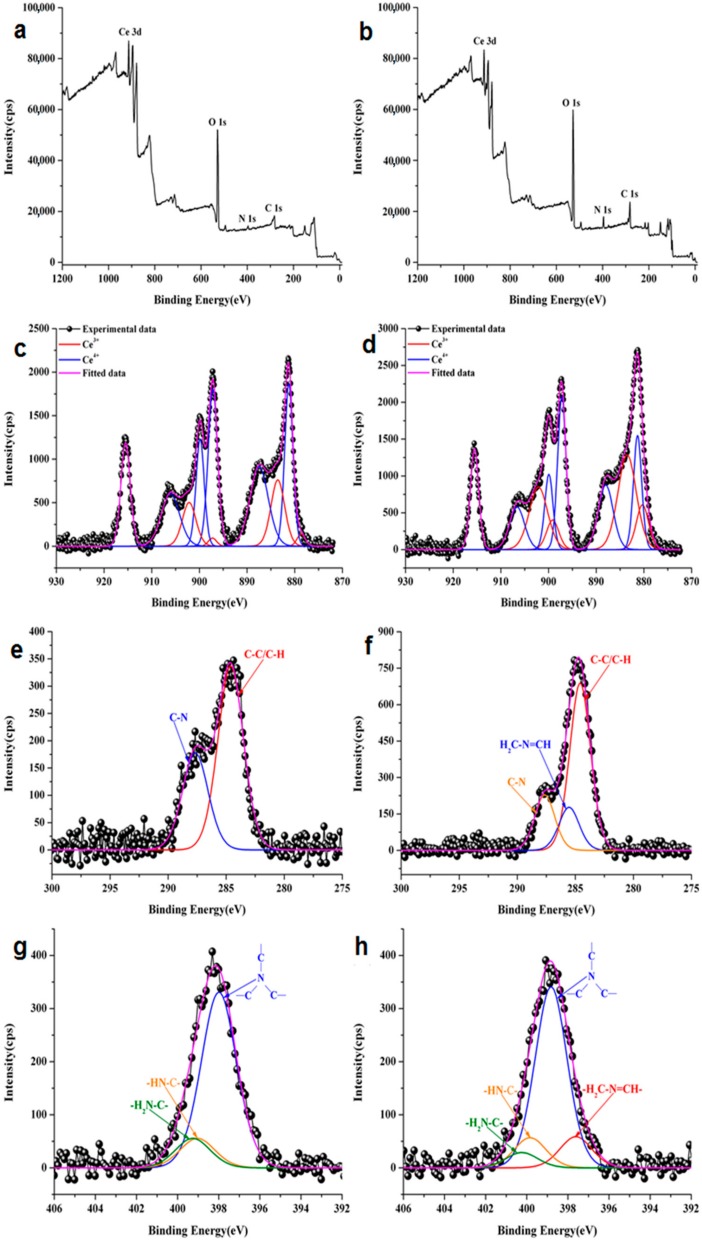
X-ray photoelectron spectroscopy (XPS) wide scan spectra for (**a**) CePEI, (**b**) CePEI-GA. Deconvoluted XPS spectra for CePEI (left) and CePEI-GA (right) specific for Ce 3d (**c**,**d**), C 1s (**e**,**f**), N 1s (**g**,**h**).

The XPS wide scan spectra of CePEI and CePEI-GA are presented in Figure 4a,b and contain all characteristic elements for the investigated conjugates.

Furthermore, the XPS analysis was also carried out to investigate the surface oxidation states of the elements, to determine the Ce^3+^/Ce^4+^ ratio and the imine bond formation present in the synthesised CePEI and CePEI-GA samples. Figure 4c,d show the typical Ce 3d XPS spectra of both synthesised cerium oxide nanoparticles. After the deconvolution, the data reveals the presence of mixed valence state of cerium with 10 characteristic peaks related to Ce^3+^ and Ce^4+^ oxidation states. 

After a close introspection on the relative concentration of Ce (Table 1) for the CePEI and CePEI-GA samples, a higher relative concentration of Ce^3+^ is found to be present in CePEI-GA conjugate (ca. 43.9%) than in the CePEI precursor (ca. 19.1%). This observation can predict that the antioxidant properties of CePEI-GA will be better than those of his CePEI homologue, as it was previously demonstrated that a good antioxidant activity of cerium oxide nanoparticles is obtained when the relative concentration of Ce^3+^ is in the 40–60% range [26]. The C 1s high resolution spectrum for the CePEI sample was solved in two characteristic peaks attributed to the C–C/C–H (284.6 eV) and C–N (287.7 eV) bonds (Figure 4e), while the one for the CePEI-GA sample shows an additional characteristic peak of the –HC=N– imine bond (285.5 eV), due to the reaction between the aldehyde groups of GA with those amine groups of PEI (Figure 4f). This is also observed in the deconvoluted N 1s peak of CePEI when three characteristic peaks specific for PEI are found: –C–N–C– (398 eV), –HN–C– (399.1 eV) and –H_2_N–C– (399.3 eV) bonds (Figure 4g). A new specific peak for the –N=CH– imine bond (397.6 eV) is observed in the CePEI-GA conjugate (Figure 4h).

### 3.6. Size and Morphology Studies

The synthetised nanoparticles are morphologically and dimensionally characterised by TEM spectroscopy and DLS methods.

In the present study, the diameters of obtained nanoparticles were evaluated by direct measurement on TEM micrographs. Hydrodynamic diameters of PEI-coated nanoparticles measured by DLS are often several times higher than those measured by TEM, which is explained by the fact that in aqueous solution, PEI is a polycation that expands and changes the apparent diameter of the solid core [6]. On the other hand, the aggregation of nanoparticles is inherent, either because of magnetic properties, or because of the aggregation of polymers that cover the nanoparticles.

**Figure 5:**
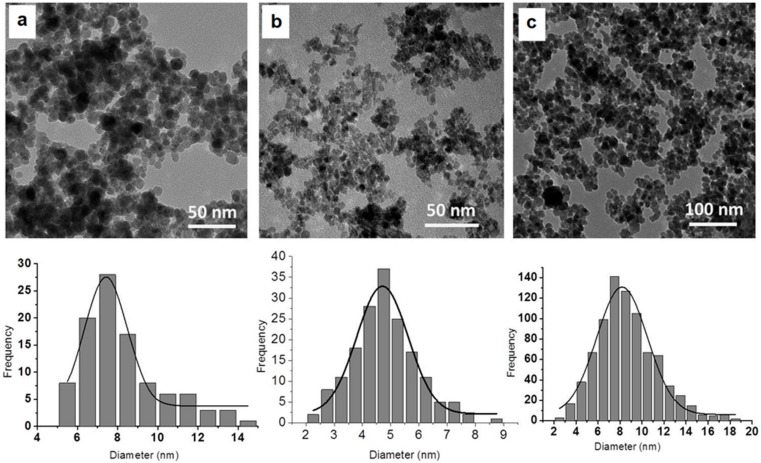
Transmission electron microscopy (TEM) micrographs of MPEI (**a**) CePEI (**b**) and MCePEI-GA (**c**) nanoparticles.

As can be seen in Figure 5, the TEM micrographs show a spherical shape with uniform size distribution for all types of studied nanoparticles, allowing mainly the visualisation of the metal oxide core, with the polymer coatings having a low influence on the nanoparticle diameters. MPEI nanoparticles (Figure 5A) present an average diameter of 7 nm, similar to our previously reported results [6]. CePEI-GA nanoparticles (Figure 5B) reveal a mean diameter of 5 nm; this low value makes them highly effective as scavengers for the ROS species present in biologic milieus [26]. Due to the repeated cross-linking reactions of coating polymers, followed by the inactivation of the free aldehyde groups with PEI, as described in the synthesis step, a slight increase in the mean diameter of the MCePEI-GA nanoparticles, about 8 nm, is observed (Figure 5c). It should be noted that the TEM image of the sample when MPEI and CePEI-GA are linked to each other through GA (MCePEI-GA) can no longer distinguish between the two types of inorganic nanoparticles, as they are embedded in a polymeric matrix. This interpretation is reasonable, given that the two types of inorganic nanoparticles in the matrix are of similar sizes, with a difference in average diameters of about 2 nm.

DLS measurements of the CePEI nanoparticles reveal an aggregation tendency, with its hydrodynamic diameter around 824 nm, which also reflects the expansion of PEI chains from the shell due to water solvation, as suggested by the positive zeta potential, with a value of +15 mV. A slight decrease in the hydrodynamic diameter (641 nm) and an increase in the zeta potential value to +31.3 mV are observed when the PEI shell is cross-linked with GA alongside the inactivation of the free aldehyde groups with additional PEI.

The hydrodynamic diameter of the bare MNP is around 490 nm, with a negative zeta potential of −24 mV at the pH value of 9, and −1 at neutral pH. Their covering with PEI leads to the preparation of MPEI nanoparticles with high positive zeta potential values, up to +43 mV, and with a hydrodynamic diameter of around 680 nm, being in line with our previously published results [6]. The variation of the zeta potential from the negative to the positive value has proven that the bare MNPs were coated with PEI.

MCePEI-GA formation leads to the obtaining of nanoaggregates with the hydrodynamic diameter of 210 nm, measured by DLS. As it was concluded by their TEM micrograph, the MPEI and CePEI are conjugated in polymeric matrices, giving them a positive zeta potential of +30 mV. In any case, positive values are also found for other nanoparticle types covered by polycations, such as chitosan [36].

### 3.7. Magnetic Properties

**Figure 6:**
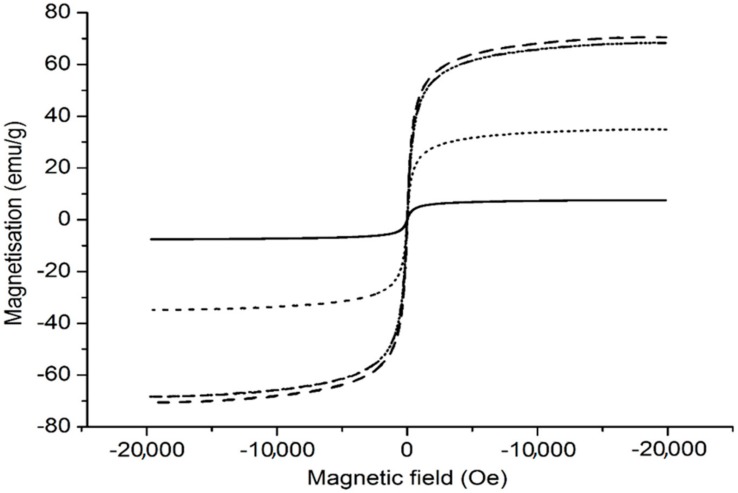
Magnetisation loops for magnetic nanoparticles (MNPs) (dash); MPEI (dash dot); physical mixture of MPEI and CePEI (dot); and MCePEI-GA (solid).

The magnetic properties for dried bare MNP, MPEI and MCePEI-GA nanoparticles were analysed using a vibrating-sample magnetometer, and the results are presented in Figure 6.

The magnetisation curves show that all the investigated samples present superparamagnetic properties, suggested by very low values of the residual magnetisation (in emu/g for MNP: 1.05; MPEI: 0.91; MCePEI-GA: 0.12) and of coercivity (in Oe for MNP: 9.74; MPEI: 6.95; MCePEI-GA: 7.41). The saturation magnetisation value of bare MNP decreases from 70.63 emu/g (Figure 6, dashed line) to 68.34 emu/g for MPEI (Figure 6, dash dotted line) and 7.55 emu/g for MCePEI-GA (Figure 6, solid line). The decrease in saturation magnetisations is due to the presence of PEI on bare nanoparticles surfaces to which are added the values of the thicknesses and densities of the polymeric shells. The results are in line with the results published in the case of MNP coated with PEI [37]. Furthermore, some authors suggest that covering bare MNP with polymers bearing functional groups may cause the decreasing of the magnetic spin mobility, leading to the reduction of collinearity and of the resulting magnetic moment [38,39]. The magnetisation value decreases from 70.63 to 68.34 emu/g for uncovered MNP and that covered with PEI, respectively, and it is in good agreement with our previous results on MNP covered with PEI [6]. Instead, a drastic reduction in MCePEI-GA magnetisation is observed, up to 7.55 emu/g, and this is mainly due by the presence of PEI in a large amount around inorganic cores, that was needed to ensure the coverage of bare nanoparticles and to inactivate the aldehyde groups resulting from PEIs cross-linking (Figure 1). In order to verify this hypothesis, a physical mixture between MNP and CePEI conjugates was performed, keeping the same proportion as in the MCePEI-GA nanoparticles syntheses. Thus, the new formed conjugate is stabilised by physical interactions, in which the influence of the GA crosslinker and the additional amount of PEI required to inactivate the aldehyde groups are eliminated. The obtained system is relatively stable, with the magnetisation value of 34.87 emu/g (Figure 6, dotted line), ranging from MPEI to MCePEI-GA, confirming the PEI’s contribution in the dramatic decreasing of magnetisation [40]. Note that the low value of magnetisation saturation does not influence biomedical applications, it being well-known that nanoparticles with magnetisation up to ~2.2 emu/g are reported as a tool for cancer treatment [41].

### 3.8. In Vitro Radical Scavenging Activity

Cerium oxide is well known in literature for its antioxidant activity, and it is reported to have mimetic enzyme activity that imitates superoxide dismutase, catalase, phosphatase, oxidase or peroxidase. This is possible due to the deficiency of oxygen atoms in the crystalline network, which makes the cerium existence in two oxidation states, Ce^3+^ and Ce^4+^ [42]. 

The antioxidant activity of the synthetised cerium oxide nanoparticles is demonstrated through DPPH assay. The method involves measuring the absorbance at 517 nm of a freshly prepared DPPH alcoholic solution, and of a solution with antioxidant and DPPH. Due to their free radical scavenging properties, the antioxidants (in our case CePEI-GA nanoparticles) inactivate DPPH radicals, which results in discoloration (from a deep violet to a pale yellow colour) and a decreased absorbance peak from 517 nm in UV spectrum. Then, using Equation (1), the percentage of inhibition for each used concentration of antioxidant can be calculated. It should be noted that the free radical scavengers or antioxidant properties are not related in our case to cerium only, being evaluated according to the total antioxidant activity of the studied nanoparticles [31,43].

**Figure 7:**
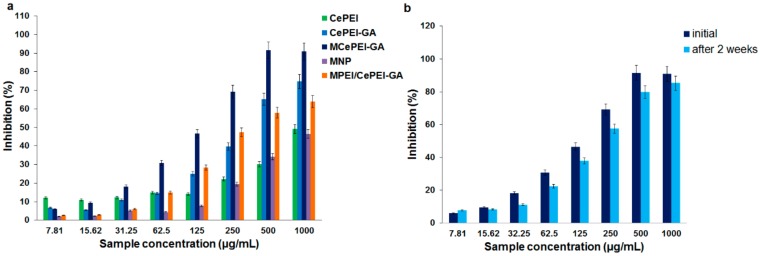
Antioxidant activity against 1,1-diphenyl-2-picryhydrazyl (DPPH) of: (**a**) CePEI, CePEI-GA, MCePEI-GA, MNP and MPEI/CePEI-GA samples; (**b**) MCePEI-GA over time.

As it can be seen from Figure 7A, the MCePEI-GA conjugate, in the 20 to 90% DPPH inhibition interval, exhibits the best antioxidant activity, even if the cerium content is lower than in the CePEI and CePEI-GA conjugates. The same rules are kept when antioxidant properties of the CePEI-GA conjugate are compared with those of CePEI. These results can be correlated with two factors which resulted from the synthesis: First, the Ce^3+^/Ce^4+^ ratio of CePEI-GA nanoparticles is higher than in those of CePEI, and second, the presence of unsaturated imine groups (as a result of the reaction between the amine groups of PEI and aldehyde groups of GA) in the cross-linked polymer coating of inorganic nanoparticles. As a result, both factors may improve the free radical scavenging properties of the nanoparticles. In Figure 7a it can be observed that MNP, MPEI and the physical mixture of MPEI and CePEI-GA (MPEI/CePEI-GA) in ratio 1:1 (w/w) exhibit noticeable antioxidant activity, which explains once more why MCePEI-GA nanoparticles possess high anti-oxidant activity.

As it can be seen from the XPS spectrum of the CePEI-GA conjugate (Figure 4d), the Ce^3+^/Ce^4+^ ratio is higher than of his CePEI homologue, which leads to the supposition that, during the PEI crosslinking process with GA, Ce^4+^ is reduced to Ce^3+^, being a catalyst in the oxidation of aldehydes in the PEI cross-linking process [44]. 

The presence of Ce^3+^ on the surface of nanoparticles is correlated with the increased number of defects caused by oxygen vacancy, which increases the area of the specific surface, leading to better scavenging activity [26]. Furthermore, the degree of DPPH inhibition is also influenced by the number of imine bonds of the studied systems [45,46]. In the case of MCePEI-GA (Figure 1), the number of cross-linking with GA is higher than in the CePEI-GA conjugate; thus, the number of imine double bonds is almost double in MCePEI-GA as compared with its CePEI-GA precursor, and as a consequence, the degree of DPPH inhibition is higher for the MCePEI-GA system.

Regarding the stability of MCePEI-GA conjugates in time, it can be seen from the Figure 7b that they present a good stability, making them good candidates for their uses in subsequent tests.

### 3.9. In Vitro Cytotoxicity Studies

**Figure 8:**
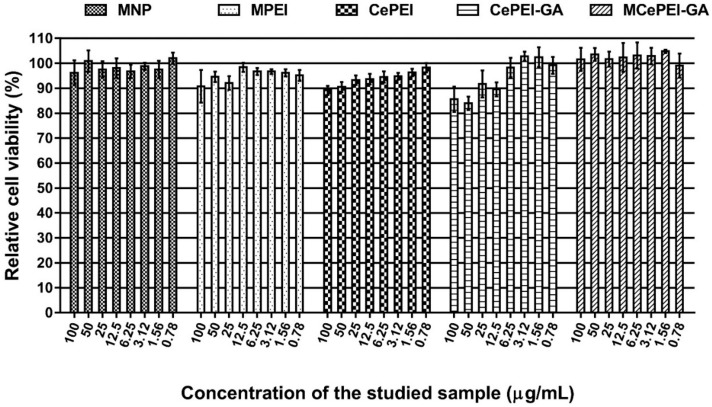
In vitro cytotoxicity assay using the (3-(4,5-dimethylthiazol-2-yl)-5-(3-carboxymethoxy-phenyl)-2-(4-sulfophenyl)-2H-tetrazolium) (MTS) method for MNP, MPEI, CePEI, CePEI-GA and MCePEI-GA nanoparticles.

The cytotoxicity of the synthesised conjugates was tested in triplicate in parallel with a control, using the MTS assay, on the NHDF cell line (Figure 8). After 48 h of incubation, the relative cell viability decreases to the limit of acceptance (90%) at high sample concentrations (100 µg sample/mL), except for those of CePEI and CePEI-GA, when at high concentrations the relative cell viability is around 80%, but still remains in the acceptance limit. The decrease in cell viability can be attributed to the presence of the Ce^3+^ and GA cross-linker in higher concentrations, and as demonstrated before, may lead to an increase in the toxicity of nanoparticles [26]. What is interesting and beneficial is that the MCePEI-GA conjugate exhibits very good cell viability compared to the control sample, even at high concentrations, perhaps due to the fact that cytotoxicity was studied in terms of the total amount of nanoconjugates, and the Ce^3+^ fraction is minimal in its composition [47].

### 3.10. In Vivo Studies

As it is well known, the overproduction of reactive radical species in the body can lead to disease. The occurrence of this phenomenon can be counteracted by the use of antioxidants. Thus, for therapeutic purposes, different antioxidants are administered, such as vitamins, vegetable extracts, or synthetic antioxidants. 

Over time, this strategy proves to be more or less effective, so it is necessary to quantify the antioxidant properties of biological fluids/organs before and after the administration of antioxidant drugs [48]. Therefore, given the antioxidant nature of the nanoparticles synthesised here, it is important to investigate their antioxidant effects by comparing the antioxidant status of living organisms before and after their administration. This can be done by the determination of the antioxidant level of various fluids and organs from the studied subjects.

**Figure 9:**
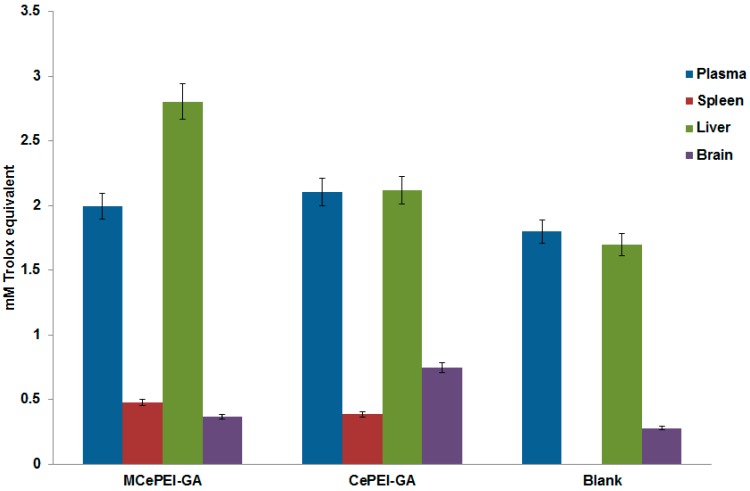
Total antioxidant capacity of the plasma, spleen, liver and brain collected from untreated and treated mice with antioxidant species.

The measurements show that all untreated and treated biological samples contain a certain quantity of antioxidants expressed in Trolox equivalents (Figure 9). Obviously, in the organs and biological fluids of the blank samples, there are naturally-occurring antioxidant activities in the series: Plasma > liver > brain, while the antioxidant activity of the spleen is almost zero. The results confirm previous published data that plasma presents the highest antioxidant content compared to other organs, due to the existence in its composition of enzymatic (SOD, catalase, glutathione peroxidases) and non-enzymatic antioxidants (glutathione reduced, vitamin A, ascorbic acid, vitamin E etc.) [49].

**Figure 10:**
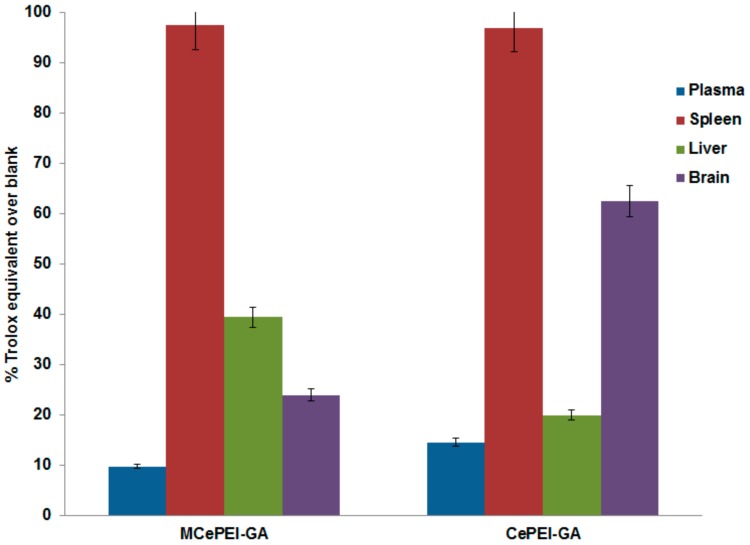
The added antioxidant percentages to the blank samples (in Trolox equivalents) in the treated samples with CePEI-GA and MCePEI-GA conjugates.

The samples collected from the mice treated with MCePEI and CePEI-GA contain more antioxidant activity than untreated ones, demonstrating the increased ability of treated mice to fight harmful active radical species in the body (Figure 10). In this context, the antioxidant activity of plasma increases with 14% and 9% for treated mice with CePEI-GA and MCePEI-GA, respectively. In the liver, the antioxidant activity increases with 20% when treated with CePEI-GA and with 39.47% when treated with MCePEI-GA, being higher than in plasma, ensuring the annihilation of a quantity of higher free radicals that exists in this organ due to its specific metabolic processes [50]. Very interesting results are obtained for spleen and brain samples treated with CePEI-GA, where the antioxidant activity increases with approx. 97% and 62.6%, respectively, while for those treated with MCePEI-GA, this increases with 97.7% and 23.95%, respectively. Apparently, the antioxidant activity exerted by the two CePEI-GA and MCePEI-GA conjugates is more efficient for the spleen; additionally, CePEI-GA is more efficient for the plasma and brain, while MCePEI-GA is for the liver.

## 4. Conclusions

The CePEI-GA cerium oxide nanoparticles synthesised by our group, with spherical morphology and a mean diameter of 5 nm (demonstrated by TEM), demonstrates free radical scavenging properties in vitro against the DPPH radical and in vivo against the ABTS^+^• cation radical due to the presence in their structures of a Ce^3+^/Ce^4+^ ratio of 43.9%/56.1%, as demonstrated by XPS. These features, together with low cytotoxicity (demonstrated on the NHDF cell line) make them good candidates for medical applications. An increased Ce^3+^/Ce^4+^ ratio is obtained after the PEI reticulation of the cerium oxide nanoparticles shell with GA aldehyde, and this is maybe due to the catalytic effect of Ce^4+^ on the oxidation of GA during cross-linking process, when Ce^4+^ is reduced to Ce^3+^. After the reticulation of the PEI shell of cerium oxide nanoparticles with GA aldehyde, the presence of imine bonds is visualised in the FT-IR and XSP spectra.

The MCePEI-GA magnetic conjugate with spherical shape and mean diameter of 8 nm presents superparamagnetic properties, allowing them to be guided under an external magnetic field to certain targets. Their structure was demonstrated using FT-IR, Raman and EDX methods. The morphology and surface properties of obtained nanostructures are determined with TEM and DLS methods. In vitro, MCePEI-GA demonstrates good biocompatibility on the NHDF cell line and a promising antioxidant activity against DPPH (around 50% for a sample concentration of 125 µg/mL), being much higher than those of CePEI and CePEI-GA nanoparticles. An increased antioxidant activity of MCePEI-GA conjugates as compared with of CePEI-GA precursors is observed, despite the fact that MCePEI-GA conjugates contain less concentrations of cerium oxide nanoparticles. The existence of a higher amount of imine bonds in the MCePEI-GA structure plays an important role for the increased antioxidant activity. Additionally, MCePEI-GA conjugates present a good stability over time. In vivo, MCePEI-GA conjugates show a good antioxidant capacity against the ABTS^+^• radical cation after their administration in mice. In this case, the added antioxidant percentages to the blank samples are given in the following series: Spleen (97%) > liver (39%) > brain (20%) > plasma (9%).

In conclusion, further extensive histology tests are needed to establish the mechanisms of action across the organs of treated mice, being necessary for the recommendation of the MCePEI-GA conjugates to be used in a living body.

## Figures and Tables

**Table 1:** Ce oxidation states obtained from the deconvolution of Ce 3d high resolution spectra.

Assignment	Binding Energy (eV)	CePEI Relative Concentration (%)	Total	Binding Energy (eV)	CePEI-GA Relative Concentration (%)	Total
Ce^3+^	878.7	1.9	19.1%	880.3	10.0	43.9%
	897.3			898.9		
	883.5	17.2		883.5	33.9	
	902.1			902.1		
Ce^4+^	881.2	25.1	80.9%	881.3	15.1	56.1%
	899.8			899.9		
	887.5	27.8		888.0	17.6	
	906.1			906.6		
	897.2	28.0		897.2	23.4	
	915.8			915.8		

## Abbreviations

MNP	magnetic nanoparticles
CeNP	cerium oxide nanoparticles
PEI	Polyethyleneimine
GA	Glutaraldehyde
MPEI	magnetic nanoparticles coated with polyethyleneimine
CePEI	cerium oxide nanoparticles coated with polyethyleneimine
CePEI-GA	cerium oxide nanoparticles coated with polyethyleneimine and reticulated with glutaraldehyde
MCePEI-GA	hybrid iron oxide—cerium oxide nanoparticles coated with polyethyleneimine and reticulated with glutaraldehyde
DPPH	2,2-diphenyl-1-picrylhydrazyl
ABTS, ABTS^+^•	2,2′-azino-bis(3-ethylbenzthiazoline-6-sulfonic acid and corresponding radical cation
NHDF	normal human dermal fibroblasts
